# Supplementary material for: Challenges and constraints of dynamically emerged source and sink in atomtronic circuits: From closed-system to open-system approaches
Source: Sci Rep. 2016 Nov 16;6:37256. doi: 10.1038/srep37256 (PMC5110959; doi:10.1038/srep37256)
Supplement: Supplementary Information [file srep37256-s1.pdf]

Supplementary information for "Challenges and constraints of dynamically emerged source and sink in atomtronic circuits: From closed-system to open-system approaches"

Chen-Yen Lai[1] and Chih-Chun Chien[1]

[1]University of California Merced, School of Natural Sciences, Merced, 95343, USA.

1 Mean Field Analysis of Equilibrium Sink

When the coupling constant U and sink-potential depth V are large compared to the hopping coefficient J, we can estimate how many bosons are allowed in a sink potential by ignoring the kinetic energy. For a system with N particles, the on-site energy of all particles localized in the sink is approximated by $E(N) \approx \frac{U}{2}N(N-1) - VN$. We construct another state where $N_{out} \ll N$ particles are outside the sink, and its energy is approximated by $E(N - N_{out})$. The condition that the bosons are more stable to stay in the sink in this approximation is $E(N) - E(N - N_{out}) < 0$, which leads to

$$\left(\frac{V}{U}\right)_c > \frac{2N - N_{out} - 1}{2}. \tag{1}$$

2 Sink in continuum model in and out of equilibrium

For the continuum model, the ground state and its dynamics may be studied by the Schrödinger equation for noninteracting atoms or the mean-field Gross-Pitaevskii equation (GPE) [1, 2] for weakly interacting bosons, as previously implemented in modeling coherent transport [3, 4]. We will analyze a simplified model where a sink corresponds to a narrow square well inside a finite box, where a dilute quantum Bose gas in the weak-interaction regime can be described by a mean-field approach [5]. At zero temperature, the condensate is described by an effective condensate wave function $\Phi(r,t)$. The evolution of the condensate wave function in an external potential $V(r,t)$ is described by the GPE:

$$\left[-\frac{\hbar^2}{2m}\frac{d^2}{dx^2} + V_{ext}(x,t) + U_l N_b |\Phi|^2\right]\Phi = i\hbar\frac{\partial}{\partial t}\Phi, \tag{2}$$

where m is the mass of the bosonic atom and N_b is the number of bosons. Here we solve the GPE with algorithms involving real- and imaginary-time propagation based on a split-step Crank-Nicolson method [6, 7], and follow Ref. [8] to normalize the wavefunction with $\int dx|\Phi(x)|^2 = 1$. The coupling constant $U_l = 4\pi\hbar^2 a_s/m$ is determined by the two-body s-wave scattering length a_s. The external potential $V_{ext}(x)$ corresponds to a narrow well and is set to simulate the equilibrium or dynamical sink. A narrow, deep trap inside an overall harmonic trap has been realized in Ref. [9], and here we idealize the situation by considering square-well potentials.

The setups and their equilibrium results are shown in Fig. 1a-1e, where the system is confined in a one-dimensional box with length L_l, which is taken as the unit of length, and the particle number $N_b = 50$. We consider a square well potential of depth V_l and width $w_l \ll L_l$ at the center or at one edge. The reason we

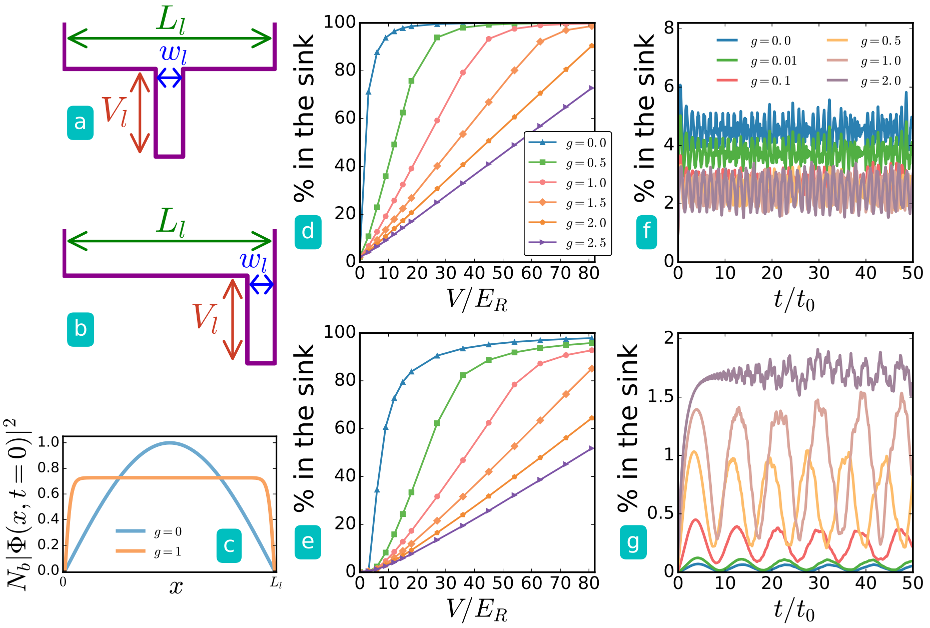

Figure 1: Illustration of the continuum model where a square-well sink is located at (a) the center and (b) the right edge of a box potential. (c) The initial density distributions with different interactions. (d) and (e): Fraction of bosons inside the sink in equilibrium as the sink depth varies. The results are from the GPE with $w_l/L_l = 0.01$ and $N_b = 25$ bosons for a sink located at (d) the center and (e) the right edge and different interaction strength g. (f) and (g): Fraction of bosons inside the sink as a function of time after a sink potential suddenly appears. The potential depth is $V_l/E_R = 9$ and the sink is located at (f) the center and (g) the right edge. In (f), the data from $g = 0.1$, 0.5, 1.0, and 2.0 are all oscillating around 2.5%. The time unit here is $t_0 = \hbar/E_R$.

explore different locations of the sink is because the initial condensate wavefunction may not be uniform and the dynamics may be different. Moreover, the initial density varies with the interaction as illustrated in Fig. 1c. When presenting the results, however, we will focus on features that are not sensitive to the location of the sink.

We choose a narrow width $w_l = 0.01L_l$ of the sink as shown in Fig. 1. For a non-interacting Bose gas at zero temperature, the number of bound states inside a square well is determined by the width and depth [10]. For weakly interacting Bose gases with coupling constant $U_l = gE_R\Omega$ in equilibrium, less particles can be accommodated in the sink with larger g due to the interaction energy, but the number of particles in the sink can be increased by increasing the depth of the sink potential. Here $\Omega = L_l^3$ and $E_R = \pi^2\hbar^2/(2mL_l^2)$ is the recoil energy.

In the adiabatic limit [11, 12] when the change of the sink potential is infinitely slow, the state remains in the ground state and the number of particles in the sink will eventually agrees with the equilibrium case. However, the time required to approximate the adiabatic limit scales as L_l^2 and hinders the scalability of the device. A similar constraint also applies to the lattice case. In the following we will focus on setups with a sudden switch-on of a sink or source. To simulate a dynamically emerged sink, the potential is uniform with $V_l(x, t < 0) = 0$ initially, then a quench to a deep sink potential leads to transport of atoms.

The suddenly emerged sink, however, does not work as expected when compared to its equilibrium counterpart. Fig. 1f shows the percentage of particles flowing into a dynamically emerged sink potential at the center, and Fig. 1g shows the case for a sink at the right edge of the system. Interestingly, in neither cases the maximal fraction of particles in the sink reaches 6%. This low effectiveness of a dynamically emerged sink is a consequence of energy conservation. The ground-state energy of the initial configuration without a sink is higher than that of the final configuration with a sink because without a sink the particles spread over the whole system while with a sink most particles tend to localize inside the sink to take advantage of the low potential

energy. In an isolated system such as cold atoms, there is no external dissipation to relax the system from the ground state of the initial Hamiltonian to the ground state of the final Hamiltonian after a sudden change of the potential. Similar phenomena where mismatches of energy spectra prohibit transport have been discussed in mass transport [13] and energy transport [14], and later on we will present similar results in the lattice case.

For the dynamically emerged sink at the center, there are less particles flow into the sink when the interaction increases, as shown in Fig. 1f. On the other hand, more particles can flow into the sink as the interaction strength increases if it is located at the edge. This subtle difference can be understood from the density distribution of the initial ground state. As the interaction becomes stronger, the density distribution of the initial ground state without a sink becomes more flat at the center and has relatively more particles towards the edge. Thus, the density at the sink potential at the edge (center) increases (decreases), as illustrated in Fig. 1c.

The ineffectiveness of a dynamically emerged sink may also be understood from the wave nature of quantum systems. It is known that when an electromagnetic wave impinges on an aperture whose diameter is much smaller than the wavelength, the transmission is severely suppressed [15]. For the atomic analogue of a sink, the particles may be viewed as matter wave whose wavelength is about the size of the whole system. The matter wave also has very low transmission into a narrow sink potential as shown in our simulations.

Since the GPE is designed for weakly interacting systems, next we will model the same dynamical process of a lattice model allowing us to analyze dynamics in the strongly interacting regime. The sink potential in the lattice model corresponds to a sudden decrease of the onsite potential on a selected site. In this approximation, there is only one bound state on the sink site for a noninteracting lattice system, so this is similar to a delta-function potential [10] in the continuum case, $V_{ext} = -V_l\delta(x)$. The delta potential only has one bound state regardless of its potential depth V_l. The physics, as one will see, is qualitatively the same as a square sink potential well in the continuum case.

2.1 Adiabatic Limit

The general solution of the time dependent Schrödinger equation at time t can be expressed as $|\psi(t)\rangle = \sum_n c_n(t)\psi_n(t)e^{i\theta_n(t)}$, where $\theta_n(t)=\frac{i}{\hbar}\int_0^t E_n(t')dt'$. By solving the Schrödinger equation,

$$\dot{c}_m(t)=-c_m\langle\psi_m|\dot{\psi}_m\rangle - \sum_{n\neq m} c_n\frac{\langle\psi_m|\frac{\partial\mathcal{H}}{\partial t}|\psi_n\rangle}{E_n-E_m}e^{i(\theta_n-\theta_m)}.$$

According to the adiabatic theorem [10], the system remains in the ground state if $\frac{\partial\mathcal{H}}{\partial t}$ is extremely small when compared to the energy level spacing divided by the natural time unit of the system which is $\hbar/E_R$ ($\hbar/J$) for the continuum (lattice) model. In the continuum model, the recoil energy is $E_R = \frac{\pi^2\hbar^2}{2mL_l^2}$ and determines the energy difference between the lowest-energy levels. The time required to reach the adiabatic limit is limited by the energy difference, so it is proportional to the square of the system size.

In Fig. 2, we show the dynamics of a small non-interacting lattice system with different ramping times. For the case with a dynamic sink potential at the center and the case with one at one edge, the sink can accommodate more particles as the ramping time becomes longer. Moreover, the results show that a dynamic sink with a longer ramping time collects more particles if the sink is at the center ($\sim 50\%$ in 2d) than at one edge ($\sim 5\%$ in 2b) under the same condition. We caution that this is again related to the initial density distribution of noninteracting bosons which is higher at the center and lower at the edge. In atomtronic applications it is more realistic to consider fast switching of the elements rather than the adiabatic limit, so our main focus is on a sudden emergent (quenched) sink or source potential.

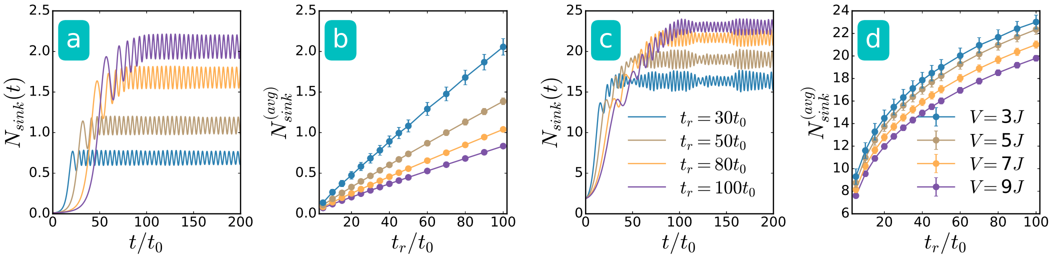

Figure 2: Non-interacting bosons in a lattice of $L = 41$ sites with $N = 40$ particles. A sink potential appears with ramping time scale t_r. (a) and (c) show the number of bosons in the sink potential versus time with sink potential depth $V = 3J$. (b) and (d) show the corresponding long-time average versus different ramping times of the sink. The sink is located at the right edge for (a) and (b), and at the center for (c) and (d). As t_r increases, the system approaches the adiabatic limit with more particles in the sink.

3 Born-Markov approximations and conserved quantities

In general, the theoretical framework of open quantum systems consists of a small system (labeled by ”s”), which may be the finite lattice considered here, and a large environment (labeled by ”e”) interacting with the system. The contribution from the environment is treated as extra terms in the equation of motion of the system. Such a composite system can be achieved by submerging a lattice system into a background of bosons, and the coupling between them can introduce dissipation or coherent cooling [16]. In such a way the system can bypass the conservation of energy. Recent advances in local heating [17] and single-site cooling [18, 19] further allow local manipulations to vary the energy of the system.

In order to describe dynamics of open quantum systems, it is more convenient to use the total density-matrix operator ρ_{total} of the system and the environment. Tracing over the environment degrees of freedom gives the reduced density matrix of the system. One usually assumes that initially the system and environment are independent, so $\rho_{\text{total}} = \rho_{\text{s}} \otimes \rho_{\text{e}}$ may be used as the initial condition. In general, the entire open quantum system cannot be solved explicitly due to the large degrees of freedom from the environment. A manageable description can be obtained with i) the Born approximation assuming that the frequency scale associated with the coupling between the system and environment is small comparing to the dynamical frequency scales of the system and environment, ii) the Markov approximation which requires that the coupling is time-independent over a short time scale and the environment can rapidly return to equilibrium without being altered by the coupling, and iii) the secular approximation which discards rapidly oscillating terms in the Markovian master equation.

The Lindblad master equation of an operator $\hat{O}$ in the Heisenberg picture [20] can be written as

$$\frac{d\hat{O}}{dt} = \frac{i}{\hbar}[\mathcal{H}, \hat{O}] + \gamma \sum_j \left[L_j^\dagger \hat{O} L_j - \frac{1}{2}\{\hat{O}, L_j^\dagger L_j\} \right]. \tag{3}$$

The observable $\hat{O}$ corresponds to a conserved quantity if it commutes with the Hamiltonian and Lindblad operators. One can see that by setting $\hat{O}$ to be the total particle number operator, it commutes with the Hamiltonian as well as the particle-number Lindblad operators and the local Lindblad operator we constructed, so the particle number is conserved in those cases.

References

[1] Gross, E. P. Structure of a quantized vortex in boson systems. *Nuovo Cimento* **20**, 454–477 (1961).

[2] Pitaevsk, L. P. VORTEX LINES IN AN IMPERFECT BOSE GAS. *Soviet Physics JETP-USSR* **13**, 451 (1961).

[3] Rab, M. *et al.* Spatial coherent transport of interacting dilute Bose gases. *Phys. Rev. A* **77**, 061602 (2008).

[4] Bradly, C. J., Rab, M., Greentree, A. D. & Martin, A. M. Coherent tunneling via adiabatic passage in a three-well Bose-Hubbard system. *Phys. Rev. A* **85**, 053609 (2012).

[5] Pethick, C. J. & Smith, H. *Bose–Einstein Condensation in Dilute Gases* (Cambridge University Press, Cambridge, 2010), 2nd edn.

[6] Muruganandam, P. & Adhikari, S. K. Fortran programs for the time-dependent Gross–Pitaevskii equation in a fully anisotropic trap. *Comput. Phys. Commun.* **180**, 1888 (2009).

[7] Vudragović, D., Vidanović, I., Balaž, A., Muruganandam, P. & Adhikari, S. K. C programs for solving the time-dependent Gross–Pitaevskii equation in a fully anisotropic trap. *Comput. Phys. Commun.* **183**, 2021 (2012).

[8] Cerimele, M., Chiofalo, M., Pistella, F., Succi, S. & Tosi, M. Numerical solution of the Gross-Pitaevskii equation using an explicit finite-difference scheme:An application to trapped Bose-Einstein condensates. *Phys. Rev. E* **62**, 1382–1389 (2000).

[9] Stellmer, S., Pasquiou, B., Grimm, R. & Schreck, F. Laser Cooling to Quantum Degeneracy. *Phys. Rev. Lett.* **110**, 263003 (2013).

[10] Kiess, H. *Quantum Mechanics* (Dover Publications, 1999).

[11] Kato, T. On the Adiabatic Theorem of Quantum Mechanics. *J. Phys. Soc. Jpn.* **5**, 435–439 (2013).

[12] Griffiths, D. J. *Introduction to Quantum Mechanics* (Pearson Education, 2005).

[13] Chien, C.-C., Gruss, D., Di Ventra, M. & Zwolak, M. Interaction-induced conducting–non-conducting transition of ultra-cold atoms in one-dimensional optical lattices. *New J. Phys.* **15**, 063026 (2013).

[14] McKay, D. C., Meldgin, C., Chen, D. & DeMarco, B. Slow Thermalization between a Lattice and Free Bose Gas. *Phys. Rev. Lett.* **111**, 063002 (2013).

[15] Jackson, J. D. *Classical electrodynamics* (Wiley, 1975).

[16] Gardiner, C. W. & Zoller, P. *Quantum Noise*. Springer Series in Synergetics (Springer Berlin Heidelberg, Berlin, Heidelberg, 2000).

[17] Brantut, J.-P. *et al.* A Thermoelectric Heat Engine with Ultracold Atoms. *Science* **342**, 713–715 (2013).

[18] Grünzweig, T., Hilliard, A., McGovern, M. & Andersen, M. F. Near-deterministic preparation of a single atom in an optical microtrap. *Nat. Phys.* **6**, 951–954 (2010).

[19] Kaufman, A. M., Lester, B. J. & Regal, C. A. Cooling a Single Atom in an Optical Tweezer to Its Quantum Ground State. *Phys. Rev. X* **2**, 041014 (2012).

[20] Breuer, H.-P. & Petruccione, F. *The Theory of Open Quantum Systems* (Oxford University Press, Oxford UK, 2002).
